# Development of an international core outcome set for treatment trials in necrotizing enterocolitis—a study protocol

**DOI:** 10.1186/s13063-023-07413-x

**Published:** 2023-05-31

**Authors:** Daphne H. Klerk, Otis C. van Varsseveld, Martin Offringa, Neena Modi, Martin Lacher, Augusto Zani, Mikko P. Pakarinen, Antti Koivusalo, Ingo Jester, Marie Spruce, Joep P. M. Derikx, Roel Bakx, Amine Ksia, Marijn J. Vermeulen, Elisabeth M. W. Kooi, Jan B. F. Hulscher

**Affiliations:** 1 Division of Neonatology, Beatrix Children’s Hospital, University Medical Center Groningen, University of Groningen, Groningen, the Netherlands; 2 Department of Surgery, Division of Paediatric Surgery, University Medical Center Groningen, University of Groningen, Groningen, The Netherlands; 3 Division of Neonatology, The Hospital for Sick Children, University of Toronto, Toronto, ON Canada; 4 Section of Neonatal Medicine, School of Public Health, Chelsea and Westminster Hospital campus, Imperial College London, London, UK; 5 Department of Paediatric Surgery, University Hospital Leipzig, University of Leipzig, Leipzig, Germany; 6 Department of General and Thoracic Surgery, The Hospital for Sick Children, University of Toronto, Toronto, ON Canada; 7 Department of Paediatric Surgery, Children’s Hospital, Helsinki University Hospital, University of Helsinki, Helsinki, Finland; 8 Departments of Paediatric Surgery, Birmingham Women’s and Children’s Hospital NHS Foundation Trust, Birmingham, UK; 9 NEC UK Charity, Nottingham, UK; 10 Department of Paediatric Surgery, UMC, Emma Children’s Hospital, Amsterdam, University of Amsterdam and Vrije Universiteit Amsterdam, Amsterdam, the Netherlands; 11 Department of Surgery, Department of Paediatric Surgery, Monastir Medical School, Fattouma Bourguiba Hospital, Monastir University, Monastir, Tunisia; 12 Care4Neo, Neonatal Patient and Parent Organization, Rotterdam, the Netherlands; 13 Department of Neonatal and Pediatric Intensive Care, Division of Neonatology, Erasmus Medical Centre, Rotterdam, the Netherlands

**Keywords:** Necrotizing enterocolitis, Delphi, Core Outcomes, Core Outcome Set, Preterm, Consensus meeting, Children

## Abstract

**Aim:**

Necrotizing enterocolitis (NEC) is the most lethal disease of the gastrointestinal tract of preterm infants. New and existing management strategies need clinical evaluation. Large heterogeneity exists in the selection, measurement, and reporting of outcome measures in NEC intervention studies. This hampers meta-analyses and the development of evidence-based management guidelines. We aim to develop a Core Outcome Set (COS) for NEC that includes the most relevant outcomes for patients and physicians, from moment of diagnosis into adulthood. This COS is designed for use in NEC treatment trials, in infants with confirmed NEC.

**Methods:**

This study is designed according to COS-STAD (Core Outcome Set-STAndards for Development) recommendations and the COMET (Core Outcome Measures in Effectiveness Trials) Initiative Handbook. We obtained a waiver from the Ethics Review Board and prospectively registered this study with COMET (Study 1920). We will approach 125 clinicians and/or researchers from low-middle and high-income countries based on their scientific output (using SCIVAL, a bibliometric tool). Patients and parents will be approached through local patient organisations. Participants will be separated into three panels, to assess differences in priorities between former patients and parents (1. lay panel), clinicians and researchers involved in the neonatal period (2. neonatal panel) and after the neonatal period (3. post-neonatal panel). They will be presented with outcomes currently used in NEC research, identified through a systematic review, in a Delphi process. Eligible outcome domains are also identified from the patients and parents’ perspectives. Using a consensus process, including three online Delphi rounds and a final face-to-face consensus meeting, the COS will be finalised and include outcomes deemed essential to all stakeholders: health care professionals, parents and patients’ representatives. The final COS will be reported in accordance with the COS-Standards for reporting (COS-STAR) statement.

**Conclusions:**

Development of an international COS will help to improve homogeneity of outcome measure reporting in NEC, will enable adequate and efficient comparison of treatment strategies, and will help the interpretation and implementation of clinical trial results. This will contribute to high-quality evidence regarding the best treatment strategy for NEC in preterm infants.

**Supplementary Information:**

The online version contains supplementary material available at 10.1186/s13063-023-07413-x.

## Introduction

Necrotizing enterocolitis (NEC) is the most lethal disease of the gastrointestinal tract of preterm infants. The peak incidence lies around 29–33 weeks after conception. NEC affects 5 to 10% of premature infants born weighing less than 1500 g [1]. Amongst defined risk factors, prematurity and birth weight are inversely related to the risk for developing NEC. The lower the gestational age, the higher the risk, with a cumulative incidence of 13–15% amongst infants born < 32 weeks of gestation. Despite efforts at prevention and treatment, overall survival has not improved over the last decades. The overall mortality rate lies between 20 and 30% and can reach 50% in surgical cases [2]. In several countries, the incidence of NEC is rising, possibly related to an increasing number of infants surviving birth at earlier gestational ages.

Management of infants with NEC is challenging. First-line treatment usually consists of nil per mouth and antibiotics. However, there is controversy around duration of nil per mouth, indications for surgery, timing of surgery, optimal surgical techniques, and the use of peritoneal drainage (PD). Common indications for surgery are bowel perforation or “clinical deterioration” despite maximal medical treatment. While there might be consensus regarding the former, the latter is less clear. Surgical options range from resection of the necrotic tissue while preserving as much bowel as possible, followed by either the construction of an ostomy or primary anastomosis when deemed feasible. A clip and drop technique with a planned relaparotomy can also be performed. Indications not to perform surgery or drain, because the infant is considered too unstable to withstand surgery, vary even more. Two earlier randomised clinical trials comparing initial PD to laparotomy failed to identify any significant differences in important outcomes. However, the results of these trials must be interpreted with caution, due to their very small sample size [3–5]. The most recent randomised controlled trial evaluating PD compared to laparotomy confirmed there was no difference in mortality or neurodevelopmental impairment at 18 to 22 months of age [6]. In this trial, infants with a spontaneous intestinal perforation (SIP) were also included. SIP can present with similar clinical and radiological symptoms as NEC, but it has a different pathophysiology and a much more favourable prognosis [7]. The pre-operative diagnosis, whether SIP or NEC, established after the study interventions were applied, was identified as a significant treatment effect modifier for both outcomes, indicating that their optimal treatment likely differs.

Both short-term and long-term morbidity resulting from NEC are significant. Short bowel syndrome can develop in around 20% of cases, even though enteral autonomy can often be redeemed through intestinal rehabilitation. Neurodevelopmental impairment is even more prevalent due to the combination of multiple factors such as brain injury following systemic sepsis, shock, and periventricular haemorrhage. In the USA, the economic burden of NEC has been estimated to be 500 million to 1 billion US dollars annually [8–10].

Though many intervention studies have been published, these are often small and report widely varying outcomes, many of which are not always relevant to scientists nor the patient and their family. This variation significantly hampers meta-analyses, as demonstrated in a recent systematic review [11]. The lack of large studies and meta-analyses complicates the establishment of evidence-based management guidelines, which may also lead to less-than-optimal outcomes for these extremely vulnerable children.

We set out to develop a NEC Core Outcome Set, thereby following the COS-STAD (Core Outcome Set-STAndards for Development) recommendations and the Core Outcome Measures in Effectiveness Trials (COMET) Handbook [12, 13]. Core Outcome Sets (COS) are groups of outcome measures, obtained using a consensus process including all stakeholders and subsequently ratified by a group of experts. A COS includes outcomes that represent the minimum to be reported in any study of the given condition. The COS for NEC treatment aims to standardise the reporting of outcomes, reduce bias, and facilitate meta-analyses. It will include the patient perspective, as one of the most important stakeholders.

## Methods

### Scope

The COS will include the most relevant outcome measures for NEC (defined as Bell’s stage ≥ 2) for all stakeholders involved in NEC, including patients and their families, from moment of diagnosis into adulthood. This COS will be designed to standardise the reporting of outcomes in NEC treatment trials, as opposed to diagnostic or prevention studies.

### Key objectives

1. Achieve consensus between stakeholders on an international COS, focused on reporting NEC outcomes in scientific literature
2. Compare outcomes prioritised by three stakeholder groups (lay, neonatal and non-neonatal panels)
3. Identify possible differences between low-middle-income and high-income countries

### Design

An international steering committee was established by contacting major research groups involved in NEC. The steering committee consists of two parent representatives, nine paediatric surgeons, four neonatologists, and two PhD candidates, from several continents. The steering committee agreed on the protocol and will provide input throughout the project. Within the steering committee, a smaller study management group (JBFH, EMWK, OCvV, and DHK) will meet regularly in between steering group meetings.

The Medical Ethics Review Board of the University Medical Center Groningen, The Netherlands, has reviewed this study and decided that this is not clinical research with human subjects as meant in the Dutch Medical Research Involving Human Subjects Act (WMO). Further ethics approval was therefore waived. With this decision, each participating country or research group will be asked to ascertain local ethical board approval or confirm that this project does not need ethical board approval. Electronic informed consent will be obtained from all participants. They will be informed about the nature of the study before voluntary registration and starting the first round. Participants will be encouraged to complete all rounds of the study but can withdraw at any moment.

**Fig. 1:**
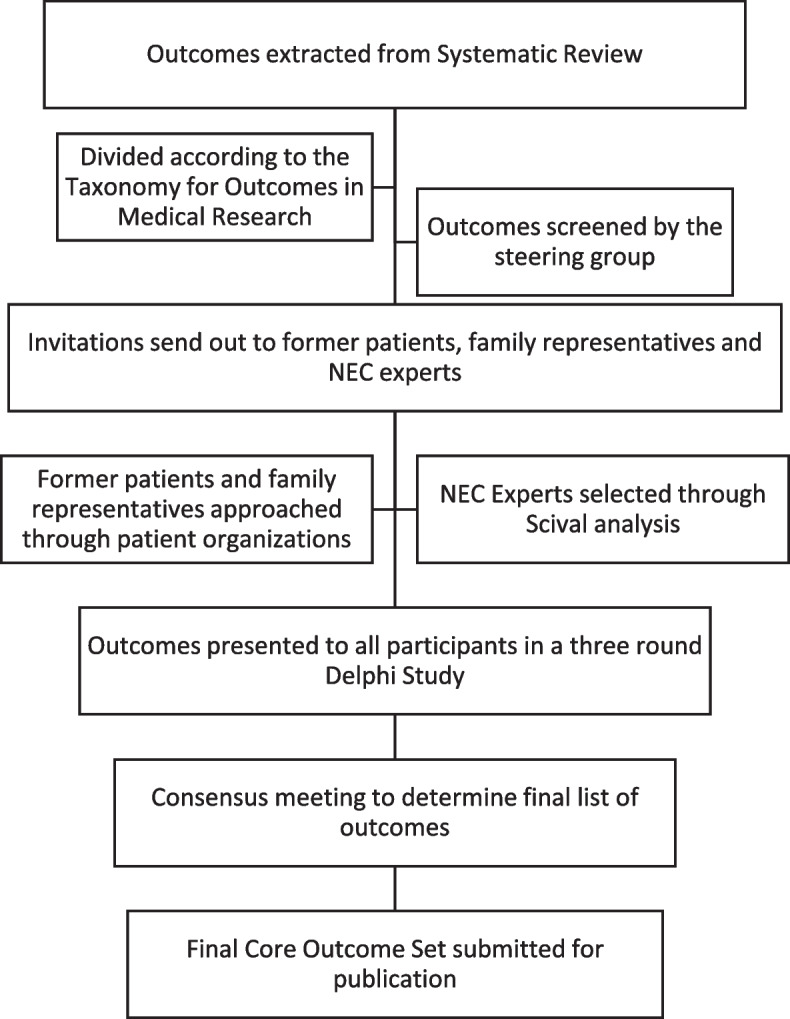
Flow chart including all steps that will be followed for developing this COS

The study was registered with the COMET initiative on 17 August 2021, study number 1920. This protocol is constructed in accordance with the COS-STAP statement; the checklist can be found in Additional file 1 [14]. Steps followed to develop this COS can be seen in Fig. 1.

#### Systematic review

We performed a systematic review, identifying all outcomes used in intervention studies and systematic reviews aimed at improvement of NEC outcomes published in the past 6 years. The review has been registered in PROSPERO (CRD42022302712) and has been submitted for publication separately. In this review, we identified sixty-four unique outcomes which we mapped to 52 outcome terms (e.g. wound infection, fistula, and stoma prolapse were mapped to ‘postoperative surgical complications’). Outcomes were subsequently excluded because of being general NICU outcomes (e.g. neonatal pneumonia or antibiotic regimen) or specific use in a treatment trial (e.g. skin burning due to use of the intervention). Ultimately, 31 were adopted into a preliminary outcomes list for the COS. The identified outcomes were categorised by two independent researchers (DHK and OCV) according to Taxonomy for Outcomes in Medical Research [15]. After review of the initial list, steering group members added fourteen terms resulting in a total of 45 candidate outcomes. A detailed list of all excluded and added outcomes can be found in Additional file 2. These outcomes will be used as the starting point for establishment of the COS with different stakeholder groups.

The steering committee and the independent Dutch neonatal patient organisation (Care4Neo) reviewed the final list of outcomes entered into the first Delphi round, to evaluate wording and add lay terms before starting the first Delphi rounds. This was done to ensure all outcomes are appropriately presented to both former patients, parents, and experts.

#### Participants

Clinicians involved in the initial treatment of NEC may have a different set of priorities compared to former NEC patients and their parents or paediatricians who treat infants at NEC follow-up consultations. To ensure that this COS represents the views of former patients and their parents, clinicians active in the neonatal period and experts involved outside the neonatal period, these stakeholders will be separated into three panels [16].

1. Lay panel—parents of infants diagnosed with NEC as well as adults who experienced NEC in infancy
2. Neonatal panel—clinicians and researchers involved in NEC management in the neonatal (NICU) period. This group will include neonatologists, (paediatric) surgeons, gastroenterologists, paediatricians, researchers, nurses, physician assistants, and fellows.
3. Non-neonatal panel—clinicians and researchers responsible for follow-up after NEC treatment, outside of the neonatal period. This group will also include rehabilitation therapists, physiotherapists, and (neuro)psychologists. If experts are involved in both the neonatal period and afterwards in the non-neonatal period, we (arbitrarily) opted to include them into the neonatal panel only.

For the lay panel, ex-NEC-patients and parents will be recruited through NEC patient organisations such as the NEC UK Charity and the Brazilian Instituto Pequenos Grandes Guerreiros, as well as through general neonatal societies such as the Dutch Care4Neo, the European EFCNI, and the Tunisian Neonatal association. Involvement of parents and patients’ representatives will be described using the GRIPP2 (Guidance for Reporting on Involvement of Patients and Public) short form checklist [17].

We will identify experts in the second and third panel using Scival Trend Analysis. Scival is a bibliometric tool that enables identification of the most prolific authors by continent. The basis for the NEC analysis is a set of publications from MEDLINE as result of a search for the MESH-term “Enterocolitis, Necrotizing” over the years 2016–2021 The set was imported into Scival for a Trend analysis, focusing on top authors and institutions in all regions of the world, looking at numbers of publications, but also at the (averaged) Field-Weighted Citation Impact (FWCI).

We will approach around 125 experts from low-middle and high-income countries. Countries are categorised based on the latest Organisation for Economic Co-operation and Development classification [18]. We will approach the top 20 authors per continent, with a maximum of one author per affiliation. In addition, the top 50 authors worldwide will be added if they were not present in their respective continental list or removed because another member of their affiliation is already present.

The invitation letters to potential participants will be developed separately for the professional stakeholder panels (neonatal and non-neonatal) and the lay panel, using appropriate language agreed on by the steering group (thereby including patient representatives). Patient organisations will also be encouraged to recruit former patients and parents or caretakers through social media. The invitation letter will include a standardised social media statement.

#### Delphi process: phase 1

We aim to include at least 40 experts divided over the two professional panels and an equal number of participants in the lay panel, for both low-middle and high-income countries. We recognise that finding former patients, parents and experts may be more difficult in low-middle-income countries. If necessary, we will invite participants from low-middle-income countries through the personal networks of steering group members. The invitation letter to all potential participants will contain a link to an online registration form in the customised Delphi Manager Tool. After confirming participation, participants will provide information on their country of work, gender, current role or function, years of work experience with NEC and amount of NEC cases encountered on average per year.

The DelphiManager software, developed and maintained by the COMET Initiative, is used to undertake the e-Delphi surveys [13]. In the first phase, participants will be presented with the list of outcomes identified through the previously conducted systematic review. Outcomes will be presented using the order of the Taxonomy for Outcomes in Medical Research, with functional outcomes presented first. Participants will score outcomes on a 9-point Likert scale, with 1–3 labelled ‘not important’, 4–6 labelled ‘important but not critical’, and 7–9 labelled ‘critical’. All participants are encouraged to keep in mind that we aim to report between 8 and 15 outcomes in the final COS. Clarifications for scientific terms including lay terms will be available in the questionnaire. As a final question in the phase 1 questionnaire, participants can add any outcomes they consider important in determining the success of NEC treatment that have not been identified in the previous outcome list. There will be no maximum to the number of additions that a participant can enter.

To limit attrition, reminder emails will be sent two weeks after the initial contact. If participants have not completed the questionnaire after 4 weeks, they will be contacted again by email to enquire if they are having difficulties in completing the questionnaire or have decided to end their participation in the study. Participants who have not completed the questionnaire within four weeks of the phase starting will be deemed not to have completed that phase and will be removed from any following phases.

The response rate from each panel group will be recorded and the median score as well as the interquartile range (IQR) will be calculated for all outcomes. Additional outcomes provided by participants will be reviewed independently by two members of the study management group to ensure they represent new outcomes and will be included in phase 2 if they were proposed by at least two participants and deemed suitable by the steering group.

#### Delphi process: phase 2

Participants who completed phase 1 will be invited to participate in phase 2. All outcomes will be carried forward to phase 2. Participants will be presented individually with their own scores and a graphical description of the distribution of scores of the three different panels. This allows participants to consider the views from their and other stakeholder groups’ before rescoring the outcomes. Subsequently, they will be asked to rescore each outcome. Participants will also be asked to score any new outcomes identified after phase 1.

Data analysis described for phase 1 will be repeated. Bias from loss of participants between phases will be assessed by looking for differences in median scores between participants who have completed both phases and participants who only completed phase 1. Any outcomes that meet the criteria of ‘consensus out’ in a minimum of two panels will be removed from the outcomes list prior to phase 3 (see the ‘consensus out’ definition below). All other outcomes from phase 2 will be carried forward to phase 3.

#### Delphi process: phase 3

Participants who completed phases one and two will be invited to participate in phase 3. The data collection process described for phase 2 will be repeated. The graphical description of the scores from all three panels is shown again, now also for the outcomes added after round 1. This allows all outcomes to undergo at least one round of stakeholder group feedback [19]. The data analysis process described for phase 2 is repeated.

#### Consensus definition in Delphi rounds

‘Consensus in’ is defined as ≥ 70% of participants rating the outcome 7–9 and ≤ 15% rating it 1–3. ‘Consensus out’ is defined as ≥ 70% participants rating an outcome 1–3 and ≤ 15% rating it 7–9. Outcomes not meeting these definitions will be classified as ‘no consensus’.

#### Final COS consensus meeting

The aim of the consensus meeting is to confirm outcomes where consensus ‘in’ has been achieved and to finalise the COS. Participants who have completed all three rounds Delphi are invited to attend an online consensus meeting. We aim to have a minimum of 20 stakeholders confirm their attendance with equally weighted panels and disciplines. Representatives from all stakeholder groups are required for the meeting to take place.

During the meeting, stakeholders are given an overview of the results of phase 3, including presentation of the top ten outcomes reaching consensus ‘in’ in the lay stakeholder group as well as the top ten outcomes consensus ‘in’ in the combined neonatal and non-neonatal stakeholder group. They will be shown how these outcomes were scored by each stakeholder panel and its consensus status. Following moderated discussion, these outcomes will be anonymously rescored using the same scoring system as the Delphi process. Outcomes that reach consensus ‘out’ or no consensus at all in phase 3 are only discussed after a unanimous decision by all attending participants. Following rescoring at the consensus meeting, outcomes reaching ‘consensus in’ will be included in the finalised COS. All others will be excluded.

### Finalising the COS

We will incorporate the OMERACT 2.0 framework in this COS and aim to include at least one outcome for every domain (death, pathophysiological or clinical manifestations, life impact, adverse events and contextual factors and resource use) [20]. Overall, we aim to develop a manageable COS, including between 8 and 15 outcomes. A separate meeting will be held to identify measurement definitions for each outcome included in the COS.

Following the online consensus meeting, a consensus document containing the final COS will be written and distributed amongst all participants. This document will be presented as appropriate during international meetings and submitted as a manuscript for publication in a peer-reviewed journal.

### Sub-group analysis

To evaluate whether the views on outcomes’ importance vary substantially between the three stakeholder groups, we will perform a sub-group analysis comparing phase 3 scores from the neonatal panel with phase 3 scores from the non-neonatal panel and the lay panel. In addition, scores from different specialties (paediatric surgeons, neonatologists, gastroenterologists etc.) will be compared. Finally, scores from participants working and/or living in low-middle-income countries will be compared with participants from high-income countries.

### Data management

Participants will enter data directly into the customised and secured DelphiManager database when they complete each questionnaire at each phase of the Delphi process. All participants will receive a unique participant number and anonymised data will be stored securely. Only members of the study management group can access this data.

## Discussion

This protocol describes a multi-phase systematic approach for developing a COS for NEC in preterm infants. We use a systematic review to identify relevant outcomes in recent NEC research to include in the first Delphi round. Currently, there is no consensus on the optimal sample size for a Delphi study. We aim to include at least 40 former patients and parents as well as 40 experts. Former NEC patients and their parents will be involved as a key stakeholder group, since their perspective might differ compared to the health professionals’ perspective. To ensure that this COS is relevant on an international level, input from three different stakeholder groups, from high- and low-middle-income countries, is essential in every step of this COS’s development. This will ensure the validity, feasibility, and promotion of the COS in future clinical trials. We do recognise that it may be difficult to reach parents from low-income countries, especially since the Delphi rounds will be in the English language. To ensure a transparent and reproducible expert selection procedure, we select experts using SciVal, guaranteeing involvement with NEC based on research output. We are aware that this may result in excluding clinicians working with NEC who are not active in NEC research. However, as the intended use of the COS is in NEC treatment trials, clinicians active in research are the intended users of this COS.

Our main limitation is that this study will not include how to measure the outcomes that are included in the final COS or at what time point the outcomes should be measured. We intend to organise a separate meeting to identify measurement definitions for each outcome included in the final COS. However, further research will likely be necessary to answer the question of timing and how to measure the presented outcomes. This core outcome set does not address how to clinically define NEC. The most frequently used definition uses the modified Bell’s criteria, including non-specific findings in Bell’s stage 1, and infants with SIP may be classified as NEC according to these criteria [21]. Other definitions have been proposed to further standardise the NEC definition, but these are not widely used yet [22]. This may be because newer definitions have better specificity, but a lower sensitivity compared to the Bell’s criteria [23]. Recent attempts were made to use artificial intelligence to define NEC and differentiate between NEC and SIP, with various algorithms accurately differentiating between NEC and SIP and performing better than traditionally used definitions [23–26]. However, all studies were done on relatively small, single-centre groups, and attempts with larger datasets are expected in the near future. We note that future studies incorporating this core outcome set need to use a uniform NEC definition and exclude infants with a post-operative diagnosis of SIP, for them to be included in a reliable systematic review or meta-analysis. This highlights the need for global consensus on this definition.

The development of this COS will help outcome data comparison and will enable adequate and efficient comparison of treatment strategies. Future trials evaluating NEC treatments will be more relevant for physicians, patients, and their parents. It will aid the interpretation and implementation of clinical trial results. This will contribute to high-quality evidence regarding the optimal treatment strategy for the management of NEC.

### Trial status

At the time of submission of this protocol, outcome selection has been completed, experts from low-middle- and high-income countries have been identified and inclusion of participants in the first Delphi round has started on 20 September 2022. The intended recruitment completion will be May 1, 2023. This protocol is version 4.0, dated 14 January 2023.

## Supplementary Information

**Additional file 1. **COS-STAP Checklist.

**Additional file 2. **Outcomes from SR.

**Additional file 3. **

## Data Availability

Not applicable.

## Abbreviations

NEC: Necrotizing enterocolitis
COS: Core Outcome Set
COS-STAD: Core Outcome Set-STAndards for Development
COMET: Core Outcome Measures in Effectiveness Trials
COS-STAR: COS-Standards for reporting
IQR: Interquartile range
