# Supplementary material for: Development of an international core outcome set for treatment trials in necrotizing enterocolitis—a study protocol
Source: Trials. 2023 May 31;24:367. doi: 10.1186/s13063-023-07413-x (PMC10230797; doi:10.1186/s13063-023-07413-x)
Supplement: Supplementary file 1 — Additional file 1. COS-STAP Checklist. [file 13063_2023_7413_MOESM1_ESM.docx]

| **Segment** | **Aim** | | **Location** |
| --- | --- | --- | --- |
| TITLE/ABSTRACT |  | | |
| Title | 1a | Identify in the title that the paper describes the protocol for the planned development of a COS | Title |
| Abstract | 1b | Provide a structured abstract | Abstract |
| INTRODUCTION |  | | |
| Background and objectives | 2a | Describe the background and explain the rationale for developing the COS, and identify the reasons why a COS is needed and the potential barriers to its implementation | 1. Introduction |
|  | 2b | Describe the specific objectives with reference to developing a COS | 2.2. Key Objectives |
| Scope | 3a | Describe the health condition(s) and population(s) that will be covered by the COS | 2.1. Scope – patients with NEC, from moment of diagnosis into adulthood |
|  | 3b | Describe the intervention(s) that will be covered by the COS | 2.1. Scope – interventions aimed at treating NEC |
|  | 3c | Describe the context of use for which the COS is to be applied | 2.1. Scope – use in NEC treatment trials, as opposed to diagnostic or prevention studies. |
| METHODS |  | | |
| Stakeholders | 4 | Describe the stakeholder groups to be involved in the COS development process, the nature of and rationale for their involvement and also how the individuals will be identified; this should cover involvement both as members of the research team and as participants in the study | 2.3. Design – identification of members of the research team.  2.3.2. Participants – identification of stakeholder groups |
| Information sources | 5a | Describe the information sources that will be used to identify the list of outcomes. Outline the methods or reference other protocols/papers | 2.3.1 Systematic Review |
|  | 5b | Describe how outcomes may be dropped/combined, with reasons | 2.3.1 Systematic Review |
| Consensus process | 6 | Describe the plans for how the consensus process will be undertaken | 2.3.3. – 2.3.7. Delphi rounds, consensus meeting |
| Consensus definition | 7a | Describe the consensus definition | 2.3.6. Consensus definition |
|  | 7b | Describe the procedure for determining how outcomes will be added/combined/dropped from consideration during the consensus process | 2.3.3. – 2.3.7. Delphi rounds, consensus meeting |
| ANALYSIS |  | | |
| Outcome scoring/feedback | 8 | Describe how outcomes will be scored and summarised, describe how participants will receive feedback during the consensus process | 2.3.3. – 2.3.7. Delphi rounds, consensus meeting |
| Missing data | 9 | Describe how missing data will be handled during the consensus process | 2.3.3. Delphi phase one |
| ETHICS and DISSEMINATION |  | | |
| Ethics approval/informed consent | 10 | Describe any plans for obtaining research ethics committee/institutional review board approval in relation to the consensus process and describe how informed consent will be obtained (if relevant) | 2.3. Design |
| Dissemination | 11 | Describe any plans to communicate the results to study participants and COS users, inclusive of methods and timing of dissemination | 2.4. Finalizing the COS |
| ADMINISTRATIVE INFORMATION |  | | |
| Funders | 12 | Describe sources of funding, role of funders | Acknowledgements |
| Conflicts of interest | 13 | Describe any potential conflicts of interest within the study team and how they will be managed | Competing interests |
