# Supplementary material for: Development of an international core outcome set for treatment trials in necrotizing enterocolitis—a study protocol
Source: Trials. 2023 May 31;24:367. doi: 10.1186/s13063-023-07413-x (PMC10230797; doi:10.1186/s13063-023-07413-x)
Supplement: Supplementary file 3 — Additional file 3. [file 13063_2023_7413_MOESM3_ESM.pdf]

University Medical Center Groningen

P.O. Box 30 001, 9700 RB Groningen, The Netherlands

Medical Ethics Review Board

Phone +31 50 361 42 04
Fax +31 50 361 43 51
E-mail metc@umcg.nl
Website metcgroningen.nl

To:
Prof. J.B.F. Hulscher, MD Ph.D.
Surgery, Child Surgery
j.b.f.hulscher@umcg.nl

Enclosure(s) ----
Ref. M22.290984

Date	01 February 2022
METc number	METc 2022/052
Title	**A Core Outcome Set for NEC.**
UMCG RR number	202200049

The Medical Ethics Review Board of the University Medical Center Groningen (METc UMCG) has discussed the above mentioned protocol and considered whether or not the research falls within the scope of the Medical Research Involving Human Subjects Act (WMO).

Based on the submitted documents the METc UMCG concludes that the above mentioned protocol is not a clinical research with human subjects as meant in the Medical Research Involving Human Subjects Act (WMO).

Therefore the METc UMCG has no task in reviewing the protocol and you do not need a WMO approval before you can start the research.

Please note that other legal Acts and/or guidelines, such as the Medical Treatment Agreement (WGBO), General Data Protection Regulation (GDPR) and codes of conduct of the FEDERA (Federation of Medical Scientific Institutions) may apply to the scientific research.

Kind regards,
on behalf of the Medical Ethics Review Board

Prof. H.P.H. Kremer, MD Ph.D.
chairman

J. Davids, MSc.
official secretary

cc: d.h.klerk@umcg.nl

umcg
